# Ultrabright Fluorescent Silica Nanoparticles for Dual pH and Temperature Measurements

**DOI:** 10.3390/nano11061524

**Published:** 2021-06-09

**Authors:** Saquib Ahmed M. A. Peerzade, Nadezhda Makarova, Igor Sokolov

**Affiliations:** 1 Department of Biomedical Engineering, Tufts University, Medford, MA 02155, USA; Saquib.Peerzade@tufts.edu; 2 Department of Mechanical Engineering, Tufts University, Medford, MA 02155, USA; Nadezda.Makarova@tufts.edu; 3 Department of Physics, Tufts University, Medford, MA 02155, USA

**Keywords:** pH sensor, temperature sensor, dual sensor

## Abstract

The mesoporous nature of silica nanoparticles provides a novel platform for the development of ultrabright fluorescent particles, which have organic molecular fluorescent dyes physically encapsulated inside the silica pores. The close proximity of the dye molecules, which is possible without fluorescence quenching, gives an advantage of building sensors using FRET coupling between the encapsulated dye molecules. Here we present the use of this approach to demonstrate the assembly of ultrabright fluorescent ratiometric sensors capable of simultaneous acidity (pH) and temperature measurements. FRET pairs of the temperature-responsive, pH-sensitive and reference dyes are physically encapsulated inside the silica matrix of ~50 nm particles. We demonstrate that the particles can be used to measure both the temperature in the biologically relevant range (20 to 50 °C) and pH within 4 to 7 range with the error (mean absolute deviation) of 0.54 °C and 0.09, respectively. Stability of the sensor is demonstrated. The sensitivity of the sensor ranges within 0.2–3% °C^−1^ for the measurements of temperature and 2–6% pH^−1^ for acidity.

## 1. Introduction

Knowledge of temperature and acidity at the nanoscale is of interest from both applied and fundamental points of view. For example, understanding the distribution of those parameters inside of the biological cell is key to understanding complex biochemical processes that are occurring in a highly heterogeneous environment of the cell. Cellular functions like gene and protein expression and protein stability are strongly temperature-dependent [1]. It is also known that cell migration, cell proliferation, wound healing [2], protein denaturation, protein folding and protein stability [3] are strongly pH-dependent. From a fundamental point of view, physical and chemical processes in the nanoscale are still not well understood in general because temperature and acidity were not measured at that scale.

Using fluorescence as an indication of pH and temperature is an attractive option because it can be accessed remotely and in three dimensions across a volume of interest. There were attempts to make a complex fluorescent molecule that would be sensitive to the change in both pH and temperature [4]. However, the fluorescence of this molecule was excessively sensitive to the changes in the ionic composition of the medium. This effectively prohibits the direct use of this molecule as a sensor. Encapsulation of sensitive molecules inside nanoparticles can decrease the dependence of the fluorescence from the medium surrounding nanoparticles, and thus, make it a sensor.

There were multiple attempts to measure both pH and temperature sequentially. For example, it was done to study biological cells, specifically, the investigation of mitochondrial acidification due to real-time monitoring of mitochondrial pH using fluorescent probes [5]. In this study, for measuring intracellular pH, the fluorescent probe was used that shows pH response based on a single peak [5]. For making measurements ratiometric, cells were post-stained with mitotracker. Measurements of pH were done by using a ratio of mitotracker and molecular probe. It was assumed that mitotracker and fluorescent probe both are homogeneously distributed. Here the concentration of mitotracker should be known and kept constant with calibration and cell measurements. The cell density on the glass-bottomed dishes for both calibration and temperature distribution measurements should be constant. Moreover, two wavelengths were used for exciting mitotracker and molecular probe. Local heat production by mitochondria inside the living cells has also been detected before using intracellular fluorescent temperature mapping [6]. Temperature mapping was performed using fluorescence lifetime imaging microscopy (FLIM), which is typically a time-consuming imaging.

Fluorescent nanoparticles changing their fluorescence spectrum as a function of acidity have previously been reported [7]. FITC, a pH-sensitive dye, and rhodamine B was used as a reference. Although rhodamine B shows a temperature dependent fluorescence, it was not discussed, and only pH measurements were demonstrated. In another study, two dyes FITC and Rb were used for measuring both pH and temperature simultaneously [8]. However, only relative changes in the pH and temperature were measured in this approach. In addition, the reported particles were of micron size, not nano. As a result, the particles have a rather limited range of applications. The fluorescent nanosensors capable of simultaneous measurements of actual accurate values of the pH and temperature have not been reported (see more comparison to the state-of-the-art in the Discussion section).

Here we report on the synthesis of fluorescent nanoparticles that can serve as nanoscopic sensors capable of measuring the values of both temperature and acidity simultaneously. The sensors are based on the encapsulation of three fluorescent dyes within the mesoporous silica matrix of nanoparticles. As was shown previously [9,10,11], such encapsulation results in obtaining ultrabright fluorescence of nanoparticles. The phenomenon of ultrabrightness was attributed to preserving the quantum yield of the encapsulated dyes while attaining a very high concentration of the dye inside the particles. The close proximity of the encapsulated dye molecules allows for the Förster’s resonance energy transfer (FRET) between the neighbor dye molecules. This allows us to build effective ultrabright fluorescent ratiometric sensors, which are excited with just one excitation wavelength. The possibility to use the FRET approach to build ultrabright fluorescent nanosensors of temperature has been recently demonstrated [12]. Our fluorescent nanosensors are ratiometric to avoid dependence on the intensity of the excitation light. This is important because it is practically impossible to control the intensity of the excitation light in an optically inhomogeneous medium. It should be noted that we previously demonstrated encapsulation of multiple organic dyes in the reported nanoparticles [13] for the purpose of multiplexed detection. To the best of the authors’ knowledge, the present work is the first report on ultrabright mesoporous silica nanoparticles for sensing both pH and temperature simultaneously.

In the presentation here, nanosensors were allowed to find the temperature and pH by using two fluorescent ratios. It should be noted that such an approach was reported for a silica-based pH and oxygen sensor [14]. Both fluorescent ratios reported in that work were independent of each other. In our approach, we do observe crosstalk between the two ratios used. To define both temperature and pH independently, we developed an algorithm that allows for decoupling the crosstalk, and as a result, minimizes the error in the definition of temperature and pH. Our algorithm allows for finding the particular wavelengths of the fluorescence spectrum to maximize the signal to noise ratio of the spectral signal. We demonstrated that using our approach, one can attain quite small errors (mean absolute deviation, or the average of residuals at different pH and temperature) of 0.54 °C and 0.09 in the definition of temperature and pH, respectively. The sensitivity of the sensor ranges within 0.2–3% for °C and 2–6% for pH^−1^. This is comparable with ranges reported in the literature [15].

## 2. Materials and Methods

### 2.1. Materials

Tetraethylorthosilicate (TEOS, ≥99%, GC, Acros Organics, Fair Lawn, NJ, USA), triethanolamine (TEA, reagent grade 98%, Sigma Aldrich, St. Louis, MO, USA), cetyltrimethylammonium bromide (CTAB, High Purity Grade, Amresco, Solon, OH, USA), ethyltriethoxysilane (ETES, 96%, Frontier Scientific, Logan, UT, USA), fluorescein isothiocyanate (FITC, Exciton, Dayton, OH, USA), rhodamine B (Rb, Exciton, Dayton, OH, USA) and Nile blue perchlorate (Nb, 95%, Sigma Aldrich) were used. RC membrane (RC membrane, Spectra/Pore, Rancho Dominguez, CA, USA) with 10–15 kDa MW was used. Deionized water was used in all synthesis.

### 2.2. Synthesis of Nanosensors

The previously reported procedure [13,16] was modified to assemble the presented pH and temperature sensor based on mesoporous silica particles. The molar ratio of chemicals in the synthesizing bath was 1 TEOS: 8.2 TEA: 0.23 CTAB: 142 H_2_O: 0.1 ETES. The ratio of FITC: Rb: Nb was 1:4:55. The mixture of TEOS (1.71 g, 8.2 mmol) and TEA (10 g, 67 mmol) was stirred for one minute and kept at 90 °C under quiescent conditions for 20 min. Another mixture of CTAB (0.69 g, 1.9 mmol), FITC (0.001 g, 0.0026 mmol), Rb (0.005 g, 0.013 mmol) and Nb (0.05 g, 0.14 mmol) and H_2_O (21 mL) was stirred for 1 min in 21 mL water and kept at 60 °C for 40 min. The CTAB, dye and water mixture were stirred at room temperature for another 15 min and kept in a cold bath for 5 min. After 5 min the mixture of TEOS and TEA was then added to the aqueous solution of CTAB and dye. ETES (130 uL, 0.8 mmol) was added after 10–15 min and stirred for another 40 min in the cold bath. After 40 min, the synthesis mixture was diluted with 30 mL water and the excess reagents were removed by dialyzing with water using the membrane of MW 10–15 kDa until no fluorescence was obtained from the dialysate (several (2–3) days). The pH of the mixture after dialysis was ~9. HCl was added to neutralize the mixture.

### 2.3. Characterization Techniques

Dynamic light scattering (DLS): DLS was used to measure the particle size and zeta potential of the nanoparticles using Zetasizer Nano ZS by Malvern Instruments Ltd., Malvern, UK. DLS uses the laser light of 633 nm and the backscattered light is monitored at an angle of 173°. The intensity-average size (Z-average) and most probable size (mean of number weighted distribution) was the average of three measurements.

Optical measurements: Cary 60 UV–Vis spectrometer (Agilent, Santa Clara, CA, USA) was used to measure the absorbance with an averaging time of 0.1 s and a scan rate of 600 nm/min. Fluorescence was measured using a Horiba Fluorelog 3 (Horiba, Japan) using a 2 nm slit width with an integration time of 0.1 s and a scan rate of 600 nm/min.

AFM imaging of sensors: An Icon Atomic Force Microscopy (AFM, Bruker, Inc., Santa Barbara, CA, USA) with NanoScope V controller with a ringing mode add-on (NanoScience Solutions, Inc., Arlington, VA, USA) was used to image the nanoparticles.

## 3. Results

### 3.1. Characterization of the Nanosensors

**Figure 1:**
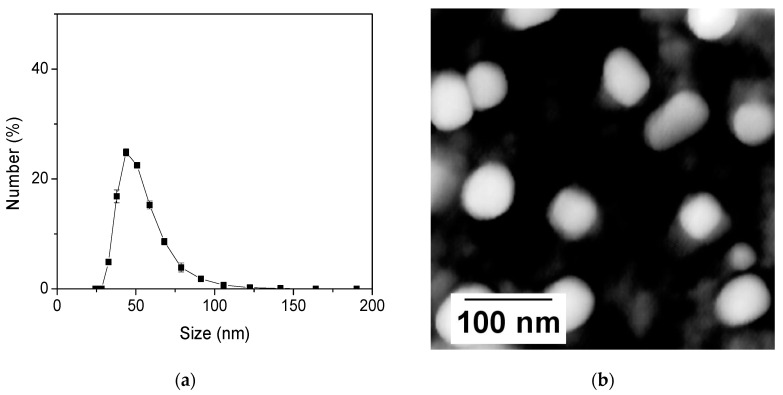
(**a**) Dynamic Light Scattering (DLS) measurements of the particle size distribution and (**b**) Atomic Force Microscopy (AFM) of nanosensors.

The DLS measurements of the particle size are shown in Figure 1a. The most probable particle size is ~51 nm. This is confirmed by direct imaging of the particles with AFM, Figure 1b. From the material point of view, the synthesized particles are identical to those previously reported by us in [11,13,16,17]. As was shown there, the internal structure of the particles is not changed when dyes are added, while the overall size of the particles can change. Thus, one can check the internal structure (measured by TEM and N_2_ absorbance) of these particles in [11,13,16,17].

**Figure 2:**
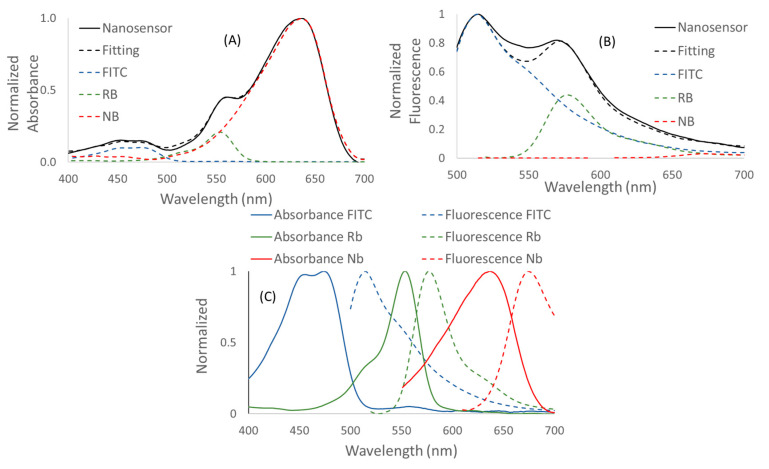
Spectral characteristics of nanosensors. (**A**) Normalized absorbance and (**B**) normalized fluorescence spectra of nanosensors. Individual component spectra of dyes used for fitting are shown. (**C**) Normalized absorbance/emission spectra of FITC, Rb and Nb dyes.

Three fluorescent dyes were encapsulated inside each nanosensor. Nile blue (Nb) was used as the reference, FITC was used for pH sensing, and rhodamine B (Rb) for temperature sensing. The spectral characteristics of synthesized nanosensors are shown in Figure 2 together with the spectral characteristics of individually encapsulated dyes. Figure 2 shows the (A) absorbance and (B) fluorescence spectra (excited at 488 nm) of the nanosensors. The dotted black line represents the fitting after demultiplexing (the addition of the individual dye components). Normalized absorbance and fluorescence spectra of individual FITC, Rb and Nb dyes are shown in Figure 2C.

The fluorescence brightness of the synthesized nanosensors was measured as described in the Equations (S15) and (S18), see reference [18] for detail. Because of the complex fluorescence composition, the brightness is calculated with respect to each of the three encapsulated dyes. The results are presented in Table 1, which shows the fluorescence characteristics of the synthesized particles, including the fluorescent brightness and quantum yield. It should be noted that the definition of the quantum yield of a fluorescent nanoparticle is somewhat ambiguous. Here we use the definition in which the quantum yield is assigned per single encapsulated dye molecule, see references [9,10] for detail. Brightness is given in the relative MESF (Molecules of Equivalent Soluble Fluorochrome) and absolute M^−1^ cm^−1^ units. MESF units relative to the corresponding dye are broadly used in flow cytometry and the characterization of particle brightness [19,20,21,22,23,24]. The absolute units are used to compare the brightness with the particles with any other fluorophore.

One can see that particles showed the brightness of ~150 and 390 relative to the free FITC and Rb dye molecules, respectively, when exciting with 490 and 550 nm. Brightness relative to Nile blue is the highest (~2000 in MESF units) when particles are excited with 630 nm wavelength. The number of FITC, Rb and Nb molecules encapsulated per particle is 180 ± 10, 390 ± 30 and 2500 ± 200, respectively. FRET efficiency and distance between dye molecules are given in Table S4 [25,26].

To find the absolute fluorescent brightness, the brightness in MESF units was multiplied by the absolute brightness of corresponding fluorescent molecules. The values of the extinction coefficient of FITC dye at 490 nm, Rb dye at 550 nm and Nb dye at 630 nm wavelength were taken from the literature, 70,000 M^−1^ cm^−1^ [27], ~100,000 M^−1^ cm^−1^ [28] and ~76,000 M^−1^ cm^−1^ [29,30], respectively. The quantum yields of free FITC, Rb and Nb dyes were used as reported in the literature 0.93 [31], 0.31 [32] and 0.27 [29], respectively. The quantum yield of the encapsulated dye was calculated in the Supplementary Information (S16) and (S17). Thus, the absolute brightness of nanosensors was (10 ± 1) × 10^6^, (12 ± 1) × 10^6^ and (40 ± 3) × 10^6^ M^−1^ cm^−1^, respectively.

The number of Nile blue dye molecules is the highest, 2500 ± 200 Nile blue dye molecules per particle. This corresponds to a 60 mM concentration of Nb dye molecules inside the particles. Thus, it corresponds to the ratio of the number of the dye molecules inside the particles FITC: Rb: Nb 1:2:14. It is interesting to compare the ratio of dyes used for synthesis, FITC: Rb: Nb 1:5:55. The difference is presumably due to the different solubility of the dyes inside the silica matrix.

**Figure 3:**
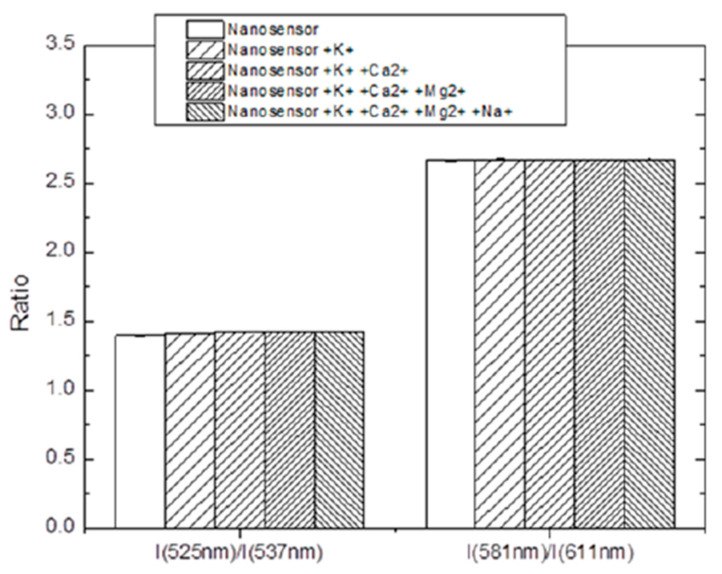
Stability of the ratios for nanosensors toward metal ions (1 mM) K^+^, Ca^2+^, Mg^2+^ and Na^+^.

When considering fluorescence sensing, it is important to verify that the fluorescence does not depend on the other parameters of the environment. In the case of the most probable use of the sensors, biomedical applications, the environment can contain complex ions, which potentially can influence the fluorescence of the encapsulated dye. Figure 3 shows the ratio of fluorescent intensities, which are used for the determination of temperature and pH (see later), in the presence of various ions typical for physiological buffers. For this purpose, the selectivity measurements were done in the presence of monovalent (K^+^ and Na^+^) and divalent (Ca^2+^ and Mg^2+^) ions. The stability of the ratios for determining pH and temperature in the presence of ions is shown in Figure 3. Ratios I(525 nm)/I(537 nm) and I(581 nm)/I(611 nm) were used for calculating temperature and pH, as shown in Figure S3. The ions K^+^, Ca^2^+, Mg^2+^ and Na^+^ were added consecutively. It can be seen that the ratios stayed virtually constant after the addition of the ions. The standard deviations across all the different addition of ions were ±0.2 °C and ±0.03 for temperature and pH, respectively.

### 3.2. Temperature and pH Calibration

**Figure 4:**
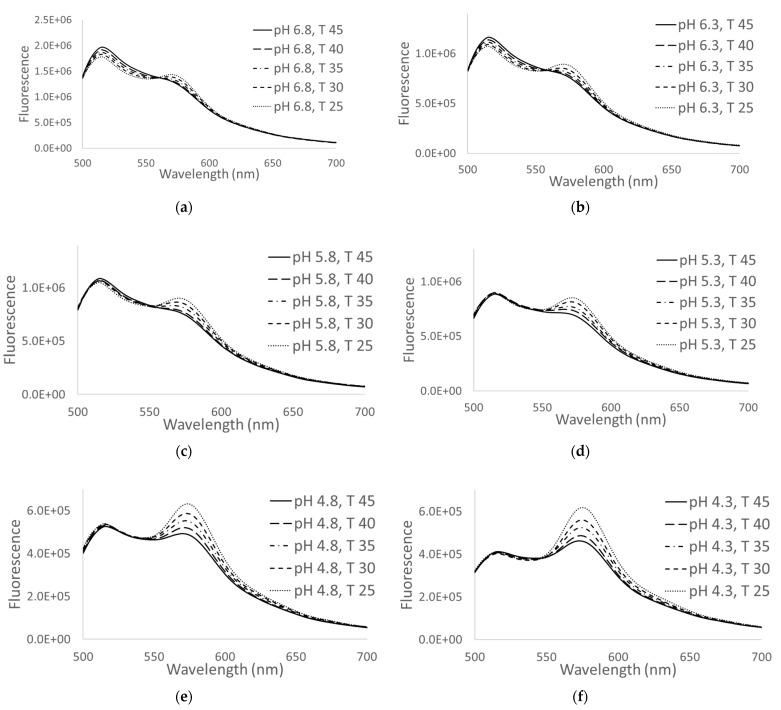
Temperature and pH responses of nanosensors to the change in temperature (shown for 25, 30, 35, 40 and 45 °C and the change in pH: (**a**) 6.8, (**b**) 6.3, (**c**) 5.8, (**d**) 5.3, (**e**) 4.8 and (**f**) 4.3, respectively. The excitation wavelength is 488 nm.

Figure 4 demonstrates the changes in the fluorescence spectral behavior of the nanosensors to the change of pH in the range of 4–7 and the temperature in the range of 25–45 °C. FITC dye is known to be sensitive to both temperature and pH while rhodamine B dye is sensitive to only temperature [8,33]. One can see that the rhodamine B peak (~570 nm) decreases with the increase in temperature, whereas the FITC peak at 515 nm decreases with the decrease of pH (increase in acidity) and increased with increase in temperature at pH 6.8 and 6.3 (Figure 4a,b). This is consistent with the reported response of FITC and rhodamine B dyes to the change of pH and temperature, respectively.

It should be noted that the presence of FRET can be seen through a relatively high fluorescence of rhodamine B and Nile blue dyes. The excitation wavelength was 488 nm. The absorbance of rhodamine B and Nile blue dyes at this wavelength is much lower compared to the absorbance of FITC. In the present configuration, FITC plays the role of the donor dye. The change of the fluorescence spectra shown in Figure 4 are due to the change of fluorescent properties of the dyes as described above, and presumably not due to the change in FRET efficiency. The latter might happen due to the change of distances between the dye molecules because of the temperature expansion of the silica matrix. However, a simple estimation shows that this effect is negligible.

The calibration of the sensors was done for a number of fixed conditions: five different temperatures (24.17, 29.63, 34.74, 39.77 and 44.8 °C) and six different acidity (pH 6.8, 6.3, 5.8, 5.3, 4.8 and 4.3). The fluorescence spectra were recorded for each of these 30 conditions. These calibration points were used to fit the polynomial functions of the algorithm as described in the next sections.

### 3.3. Method of Simultaneous Measurements of Temperature and Acidity

As one can see in Figure 4, the combination of encapsulated dyes shows a rather nontrivial dependence on both temperature and pH. There is substantial crosstalk between the responses to the change of temperature and pH. Here we present an algorithm, which allows us to decouple this complex behavior of the fluorescent spectra to define the temperature and acidity separately and with relatively high precision. We demonstrate that it is sufficient to have two ratios of fluorescence intensities at different wavelengths to determine the temperature and acidity quite accurately. To do it, first, we will find the optimal values of the wavelengths to be used to calculate the intensity ratios. Then we suggest simple polynomial functions to determine the temperature and acidity using those ratios.

### 3.4. Finding the Optimal Values of Wavelengths to Measure Temperature and Acidity Simultaneously

To define both pH and temperature in a ratiometric manner, two different fluorescence ratios corresponding to two different wavelengths should be chosen. Our approach is based on Equation (1) minimization of the ambiguity of finding both pH and temperature (due to existing crosstalk between responses to the change in pH and temperature) and Equation (2) minimization of the error of the measurements (because the fluorescence intensities have different signal-to-noise ratio at different wavelengths). To minimize the complexity of such an analysis, it is preferable to have a linear relationship between the sought ratios and pH and temperature. To find the linear relations, regression analysis was used [34]. The Pearson correlation coefficient r indicates the proportion of variance in variable Y represented by a linear function of X. This coefficient is given by the following formula:

(1) $$r^{2}=\frac{S_{\mathrm{xy}}^{2}}{S_{x}S_{y}}=\frac{{\left(\sum_{i=1}^{n}\left(X_{i}-\;\bar{X})(Y_{i}-\;\bar{Y}\right)\right)}^{2}}{\sum_{i=1}^{n}{\left(X_{i}-\;\bar{X}\right)}^{2}\sum_{i=1}^{n}{\left(Y_{i}-\;\bar{Y}\right)}^{2}},$$

where, S_xy_ is the covariance of X and Y, S_x_ is the standard deviation of X, S_y_ is the standard deviation of Y. The linear relation implies this coefficient to be close to 1.

Combining the standard deviation of pH and the ratio R and covariance of pH and R, as given in Equations (S2), (S3) and (S5), one can find the Pearson correlation coefficient for pH as follows:

(2) $$r_{\mathrm{pH}}^{2}=\frac{S_{\mathrm{pH},R}^{2}}{S_{\mathrm{pH}}S_{R}}r_{\mathrm{pH}}^{2}=\frac{S_{\mathrm{pH},R}^{2}}{S_{\mathrm{pH}}S_{R}}=\frac{{\left(\sum_{{\mathrm{pH}}_{1}}^{{\mathrm{pH}}_{n}}\left(\left(\mathrm{pH}-{\mathrm{pH}}_{\mathrm{mean}}\right)\left(\sum_{T_{1}}^{T_{n}}\left(\frac{R}{n_{T}}\right)-\;\bar{R}\right)\right)\right)}^{2}}{\sum_{{\mathrm{pH}}_{1}}^{{\mathrm{pH}}_{n}}{\left(\mathrm{pH}-{\mathrm{pH}}_{\mathrm{mean}}\right)}^{2}\sum_{{\mathrm{pH}}_{1}}^{{\mathrm{pH}}_{n}}{\left(\sum_{T_{1}}^{T_{n}}\left(\frac{R}{n_{T}}\right)-\;\bar{R}\right)}^{2}},$$

Similarly, combining the standard deviation of T, R and covariance of T and R given by Equations (S6), (S7) and (S9), one can find the Pearson correlation coefficient for temperature T given by the following formula:

(3) $$r_{T}^{2}=\frac{S_{T,R}^{2}}{S_{T}S_{R}}=\frac{{\left(\sum_{T_{1}}^{T_{n}}\left(\left(T-T_{\mathrm{mean}}\right)\left(\sum_{{\mathrm{pH}}_{1}}^{{\mathrm{pH}}_{n}}\left(\frac{R}{n_{\mathrm{pH}}}\right)-\;\bar{R}\right)\right)\right)}^{2}}{\sum_{T_{1}}^{T_{n}}{\left(T-T_{\mathrm{mean}}\right)}^{2}\sum_{T_{1}}^{T_{n}}{\left(\sum_{{\mathrm{pH}}_{1}}^{{\mathrm{pH}}_{n}}\left(\frac{R}{n_{\mathrm{pH}}}\right)-\;\bar{R}\right)}^{2}},$$

**Figure 5:**
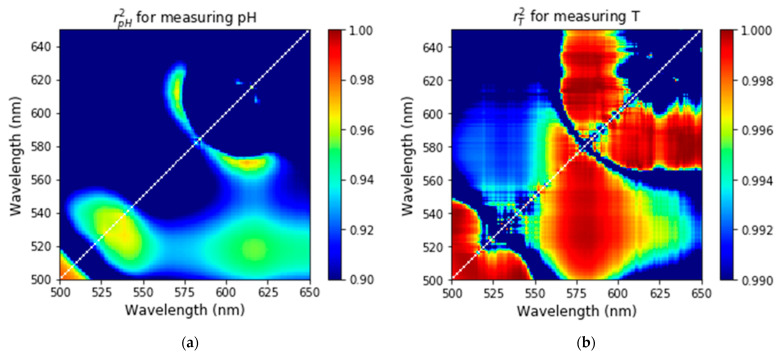
The Pearson correlation coefficients are shown for different wavelengths used to calculate the ratio of fluorescent intensities. The dependence between the model parameters (temperature and pH) and the corresponding ratios is close to linear when the Pearson coefficient is close to 1. The results are shown for pH (**a**) and temperature (**b**) measurements.

Figure 5 shows the plots of the values of the Pearson coefficients for pH and temperature calculated using the fluorescence spectra shown in Figure 4. Using Equations (2) and (3), the Pearson coefficients are calculated for different wavelengths used to calculate the ratio of fluorescent intensities. One can see that Figure 5a,b shows linearity (the Pearson coefficients close to 1) for both pH and temperature for a wavelength range of 500–550 nm. The range of 550–650 nm is also highly linear with respect to the change of temperature with the Pearson coefficient >0.996 (Figure 5b). Comparing Figure 5a,b, one can see that the ratio dependencies are more linear for temperature ($r_{T}^{2}$ > 0.996) compared to pH ($r_{\mathrm{pH}}^{2}$ > 0.95). This is in agreement with previously reported FITC-based pH sensors, which are known to be nonlinear with respect to the change in pH [35]. The regions of wavelengths, in which the dependence of temperature and pH on the corresponding fluorescense ratios is quite linear are considered further to choose the wavelengths for the ratio to obtain the minimum error of measurements.

Although we see the linear relation between the parameters, pH and temperature, and the intensity ratios, it is instructive to add a few nonlinear polynomial terms because the Pearson coefficient is not exactly 1, which indicates it may be small, but still a deviation from linearity. In addition, it will help us to verify that the nonlinear terms are small. Hereafter, we suggest the Equations (4)–(9) comprising the polynomial of the third order and a linear crosstalk product of two ratios:

(4) $$T\left(R_{T},R_{\mathrm{pH}}\right)=\;\mathrm{At}\times {\left(R_{\mathrm{pH}}\right)}^{3}+\mathrm{Bt}\times {\left(R_{\mathrm{pH}}\right)}^{2}+\mathrm{Ct}\times R_{\mathrm{pH}}+\mathrm{Dt}\times R_{T}\times R_{\mathrm{pH}}+\mathrm{Et}\;,$$

(5) $$\mathrm{pH}\left(R_{T},R_{\mathrm{pH}}\right)=\;\mathrm{ApH}\times {\left(R_{\mathrm{pH}}\right)}^{3}+\mathrm{BpH}\times {\left(R_{\mathrm{pH}}\right)}^{2}+\mathrm{CpH}\times R_{\mathrm{pH}}+\mathrm{DpH}\times R_{T}\times R_{\mathrm{pH}}+\mathrm{EpH},$$

where At, Bt, Ct, Dt Et, ApH, BpH, CpH, Dt and Et are constants, R_pH_ and R_T_ are ratios of fluorescence intensities of the chosen reflexes (i.e., R_pH_ = I(λ1)/I(λ2) and R_T_ = I(λ3)/I(λ4)).

Now we can focus on maximizing the signal-to-noise ratio of the fluorescence signal that is used to find temperature and pH, i.e., the ratio of the fluorescence intensities. It is useful to simultaneously maximize the sensitivity to the change of temperature and pH. Therefore, we suggest maximizing the signal-to-noise ratio of the following signal:

(6) $$S\left(A,B\right)=\left|\frac{\left({\left(\frac{I_{1}}{I_{2}}\right)}_{T1}-{\left(\frac{I_{1}}{I_{2}}\right)}_{T2}\right)}{\left(\frac{{\left(\frac{I_{1}}{I_{2}}\right)}_{T1}+{\left(\frac{I_{1}}{I_{2}}\right)}_{T2}}{2}\right)}\right|=\left|\left(\frac{\left(A-B\right)}{\left(\frac{A+B}{2}\right)}\right)\right|,$$

where I_1_ and I_2_ are the fluorescence intensities at wavelength 1 and 2, respectively, A and B are the ratio of intensity at temperatures T_1_ and T_2_, respectively.

According to the formula of error propagation, the error in signal S (A,B) is given by

(7) $$\delta S\left(A,B\right)=\sqrt{{\left(\partial_{A}S\left(A,B\right)\right)}^{2}{\left(\delta A\right)}^{2}+{\left(\partial_{B}S\left(A,B\right)\right)}^{2}{\left(\delta B\right)}^{2}},$$

(8) $$=\sqrt{{\left(\partial_{A}\left(\frac{\left(A-B\right)}{\left(\frac{A+B}{2}\right)}\right)\right)}^{2}{\left(\delta A\right)}^{2}+{\left(\partial_{B}\left(\frac{\left(A-B\right)}{\left(\frac{A+B}{2}\right)}\right)\right)}^{2}{\left(\delta B\right)}^{2}}=4\sqrt{\frac{B^{2}{\left(\delta A\right)}^{2}+A^{2}{\left(\delta B\right)}^{2}}{{\left(A+B\right)}^{4}}},$$

where,

(9) $$\delta A\left(I_{1},I_{2}\right)=A\sqrt{{\left(\frac{{\delta I}_{1}}{I_{1}}\right)}^{2}+{\left(\frac{{\delta I}_{2}}{I_{2}}\right)}^{2}},\;\delta B\left(I_{1},I_{2}\right)=B\sqrt{{\left(\frac{{\delta I}_{1}}{I_{1}}\right)}^{2}+{\left(\frac{{\delta I}_{2}}{I_{2}}\right)}^{2}}$$

Similar formulas can be derived for the signal defining pH.

**Figure 6:**
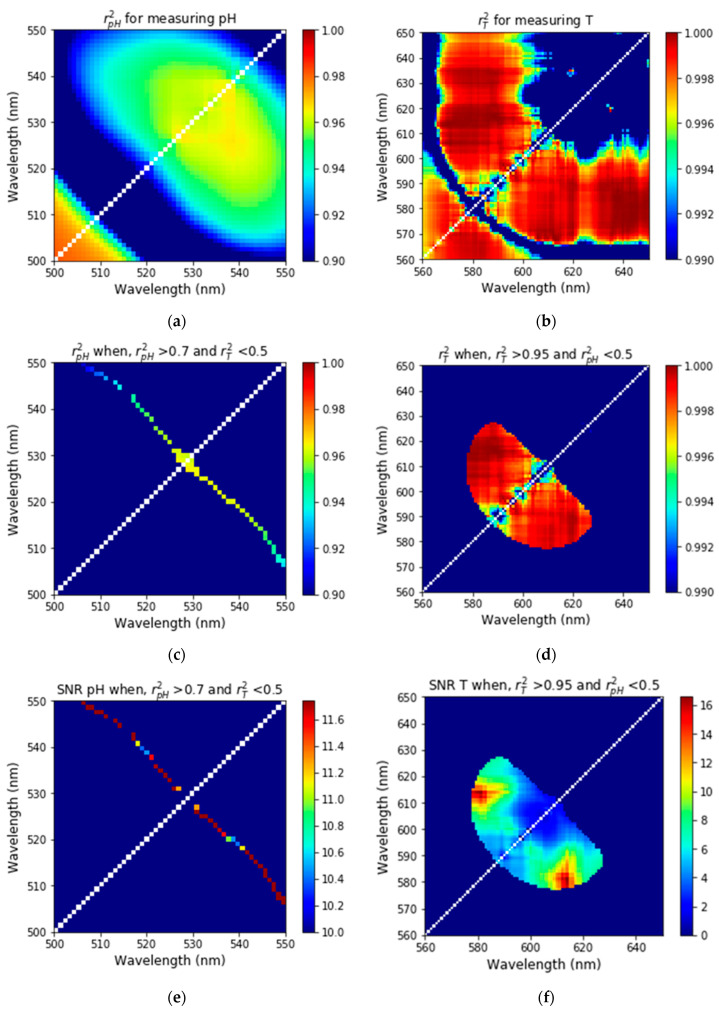
The Pearson correlation coefficient and signal-to-noise ratios for different wavelengths. The Pearson correlation coefficients for pH (**a**) and temperature (**b**) measurements. Pearson correlation coefficient for (**c**) pH when PCC of pH is greater than 0.7 while PCC of temperature is less than 0.5 and for (**d**) T when PCC of temperature is greater than 0.95 while PCC of pH is less than 0.5. SNR for (**e**) pH when PCC of pH is greater than 0.7 while PCC of temperature is less than 0.5 and for (**f**) T when PCC of temperature is greater than 0.95 while PCC of pH is less than 0.5.

Pearson correlation coefficient (PCC) and signal-to-noise ratios (SNR), S(A,B)/δS(A,B) calculated for the fluorescent nanosensors are shown in Figure 6 and Figure S2. One can see still a sufficiently broad range of wavelengths to choose from when PCC is close to 1. The freedom of choice of the optimal wavelengths can further be reduced by finding the regions of the wavelengths in which the Pearson correlation coefficient is sufficiently close to 1 and the signal-to-noise ratio is sufficiently high. In addition, to reduce the ambiguity and cross-correlation between pH and temperature, one ratio should depend on only one parameter and should be independent of the other parameter. For finding the two ratios, where one ratio depends on only one parameter and is independent of the other, the region of interest was identified such that the ratio of two wavelengths have high linearity (or dependence) to pH and low linearity (or independence) for temperature, and similarly, the region of interest for temperature was identified such that the second ratio of other two wavelengths has high linearity (or dependence) to temperature and low linearity (or less dependence) to pH. Specifically, we find two wavelengths for pH such that the PCC of pH was taken greater than 0.7 while that of temperature was taken less than 0.5 (Figure 6c). Figure 6e shows the SNR of pH for the region of interest where PCC of pH is greater than 0.7 while PCC of temperature is less than 0.5. Similarly, for finding two wavelengths for temperature, for PCC of temperature were set such that the PCC of temperature was taken greater than 0.95 while that of pH was taken less than 0.5 (Figure 6d), and Figure 6f shows the SNR of temperature for the region of interest where PCC of temperature is greater than 0.95 while PCC of pH is less than 0.5. The ratio of intensities at wavelength 582 to 614 nm showed high SNR and PCC for temperature while the ratio of intensities at wavelength 523 to 536 nm showed high SNR and PCC for pH. Hence R_T_ and R_pH_ were taken as the ratio of intensities at wavelength 582 to 614 nm and 523 to 536 nm, respectively.

### 3.5. Algorithm for Further Finding the Optimal Values of Wavelength

**Figure 7:**
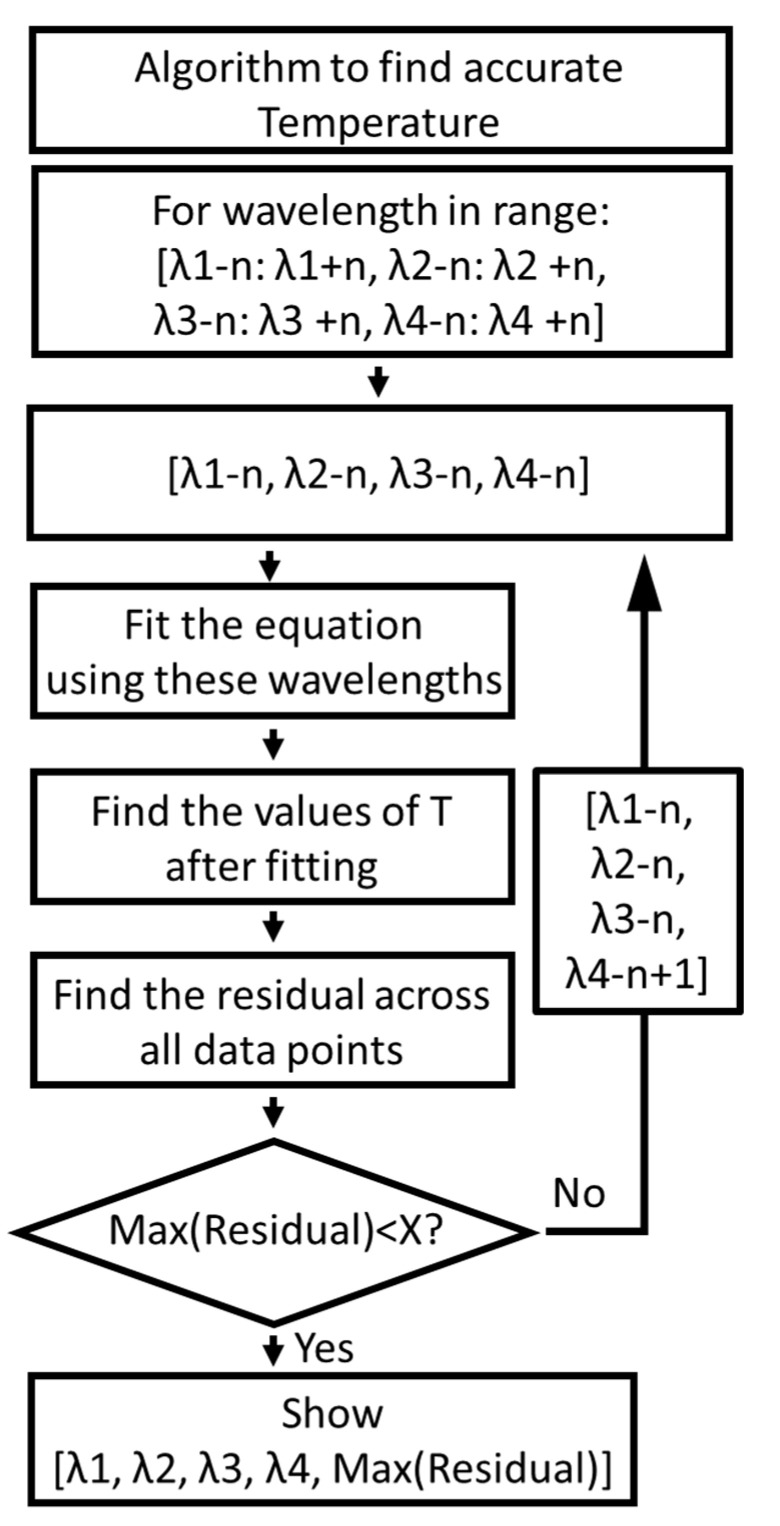
Flowchart outlining the procedure for obtaining four wavelengths for temperature. Note that the fitting for temperature was performed using Equation (4) and R_pH_ and R_T_ represent I(λ1)/I(λ2) and I(λ3)/I(λ4), respectively; X represents 1.2 °C for temperature.

In principle, the accuracy of the measuring temperature and acidity could further be improved using the algorithm shown in Figure 7. According to the algorithm, for the wavelengths in the range of λ1 − n to λ1 + n, λ2 − n to λ2 + n, λ3 − n to λ3 + n and λ4 − n to λ4 + n, the first four wavelengths (i.e., λ1 − n, λ2 − n, λ3 − n and λ4 − n) were chosen and fitted to Equation (4) and values of temperature were determined. The residual (the absolute difference between the actual values of temperature and the values measured using our nanosensors) was calculated for 30 data points (i.e., five data points for different temperatures and six data points for different pH) and the maximum of all the residuals were calculated. If the maximum residual was less than 1.2 °C for temperature, the four wavelengths and their residual were stored and displayed, and if it was greater then, one of the wavelengths was iterated and the loop continued until all the range of the wavelengths were iterated (i.e., until λ1 + n, λ2 + n, λ3 + n and λ4 + n). In this way, the four wavelengths that show a maximum residual of less than 1.2 °C were identified. For finding temperature, since the wavelengths determined using PCC and SNR were 523, 536, 582 and 614 nm for λ1, λ2, λ3 and λ4, respectively, the range of the wavelengths used was 519–527 nm for λ1, 532–540 nm for λ2, 578–586 nm for λ3 and 610–618 nm for λ4 (considering n = 4). The range was four wavelengths below and above the wavelengths determined using regression and SNR analysis. Since the temperature error should be less than 1.2 °C, the expected residual in temperature was chosen to be less than 1.2 °C. Out of all the wavelengths, the wavelengths 525, 537, 581 and 611 nm showed the lowest maximum residual of ~1.1 °C for temperature. Using the same four wavelengths, the maximum residual for pH was determined to be equal to 0.3.

Table S1 shows the ratios at different pH and temperature. These ratios were further used for multiparametric fitting. From Figure S1, it can be seen that the ratios change repeatably when pH is constant and the temperature is changed and also vice versa. This makes the pH and temperature sensor repeatable.

### 3.6. Multiparametric Fit for pH and Temperature

**Figure 8:**
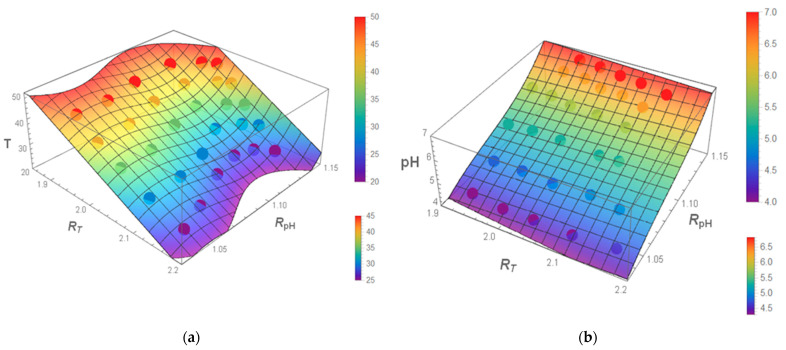
The results of the multiparametric fitting of experimental data (shown by dots) using Equations (4) and (5) to measure measuring temperature and pH, respectively. The measurements of the (**a**) temperature and (**b**) pH as a function of two ratios, R_T_ and R_pH_.

Using the wavelengths that were found, we fit the experimental data (shown in Figure 4 for a set of different temperatures and pH) with the help of Equations (4) and (5). Table 2 shows the fitted coefficients used in Equations (4) and (5). One can see that the coefficients corresponding to the nonlinear terms are indeed rather small. Both the results of the fitting and the experimental data are shown in Figure 8. One can see a rather good fit.

To estimate the errors of the measurements of temperature and pH using the functions of fitted Equations (4) and (5), we conducted four independent measurements of fluorescence at temperature 24.17, 29.63, 34.74, 39.77 and 44.8 °C and pH 6.8, 6.3, 5.8, 5.3, 4.8 and 4.3. Measuring multiple spectra and using the error propagation (see the Supplementary Information), we find the errors of measurements of both temperature and pH. The examples are shown in Table S2 (assuming fixed pH) and Table S3 (assuming fixed temperature). Corresponding ratios R_T_ and R_pH_ are shown in the Supplementary Information.

Table S1 shows the ratios that are calculated using the intensities at wavelengths 525 to 537 nm, and 581 to 611 nm wavelengths for pH and temperature, respectively. The accuracy determined using this formula was 98.4% and 98.36% for pH and temperature, respectively.

## 4. Discussion

Here we discuss the results obtained in comparison with the current state-of-the-art. Because the achievement reported is based on multiple pillars, we describe each of them separately below.

### 4.1. Use of Mesoporous Silica Versus Solid Silica as the Material of the Nanothermometers

Gold–FITC encapsulation of dye molecules in solid silica matrix has been previously reported. For example, silica nanoparticles had physically encapsulated rhodamine B dye while the silica shell was modified with APTES followed by conjugation with FITC [7]. This post-conjugation of silica particles with APTES and further attachment with FITC is not only an additional and time-consuming process, but it also reduces the quantum yield of the encapsulated dye molecules. The particles obtained served as a pH sensor due to the presence of pH-sensitive FITC dye, whereas rhodamine B served as a reference [7]. Although rhodamine B is temperature-sensitive, no temperature measurements were reported for that work because there was no third reference dye involved (a third dye is needed to make the sensor ratiometric). In another paper, a ratiometric pH sensor using FITC and Nile blue was reported [36]. Nile blue was used as a reference. FITC and Nb were conjugated on the surface of carbon dots. However, ratiometric pH and dual temperature sensors based on two ratios (three dyes) have not been made before.

The use of mesoporous silica particles as hosts for dye molecules creates a novel possibility to create ratiometric fluorescent sensors coupling two different dyes with FRET [12,13]. For extending the application of sensors to understand cellular processes, it is important that the sensors can operate at the nanoscale. Physical encapsulation of dyes in mesoporous silica could be used for encapsulating multiple dyes that are sensitive to different parameters (for example, pH and temperature in this case) in a single nanoparticle. In this work, sensor-based on ultrabright mesoporous silica nanoparticles is synthesized incorporating three dyes. Herein, FITC and rhodamine B were used for pH and temperature sensing, respectively, while Nile blue was used as a reference. This is the first demonstration of pH and temperature nanosensors based on the physical encapsulation of three dyes. The particles reported here are free of the issues mentioned above and can be used as sensors.

### 4.2. The Problem of Ambiguity of Simultaneous Measurement of Temperature and Acidity

Gold–FITC based nanoclusters have been reported for pH and temperature sensing [37]. This dual sensor was built using two fluorescence peaks coming from gold and FITC. However, for unambiguous determination of both pH and temperature, the magnitude of the intensity ratio or the initial values of pH and temperature should be known. In other work, a pH and temperature sensor was made using a co-assembly of temperature-sensitive polymer PNIPAM, pH-sensitive polymer PNIPAM-co-PAA, photoluminescence sources europium and quaternary ammonium tetraphenyl ethylene derivatives [38]. However, simultaneous temperature and pH measurements were not conducted due to the ambiguity of cross-correlation between the fluorescence dependence of the temperature and pH. The temperature measurements were performed at a constant pH 7 only. As we demonstrated in the present work, we use two different fluorescence ratios to avoid the need of the above ad hoc knowledge and solve the problem of cross-correlation.

### 4.3. Multiparametric Sensor

The other pillar used in this work is the use of multiparametric algorithms, in particular, the use of polynomial sensing Equations (4) and (5). Ideally, in a dual sensor, the two parameters should be independent of each other. Linear multiparametric sensors, a polynomial of the order of one, have been studied in [39]. Nonlinear polynomial fit for calibrating pH sensor is reported [35]. However, to the best of our knowledge, a nonlinear polynomial fit for sensing both pH and temperature has not been previously reported. In addition, the multiparametric sensor, in which the two parameters are related to each other, has not been previously reported. In this study, a nonlinear multiparametric sensor in which the two parameters are cross-correlated but avoid ambiguity in measuring both parameters (temperature and pH) is reported.

### 4.4. Stability of the Sensor

Because of a potential concern of leakage of the encapsulated dye and photobleaching, it is important to demonstrate that the sensor is stable and the readings are repeatable. The repeatability of the described sensors is shown in Figure S1. Fluorescent spectra from nanosensors were measured at pH 7 for temperatures 35 and 50 °C and, similarly, at pH 4 for temperatures 35 and 50 °C for four cycles of temperature. The errors defined as the standard deviation across four measurement cycles are shown in Figure S1. One can see that the error to signal ratio is less than 1%, Table S5.

Spectral stability with respect to the presence of different ions in the solution is shown in Figure 3 and Figure S3. Measurements were done in the presence of monovalent (K^+^ and Na^+^) and divalent (Ca^2+^ and Mg^2+^) ions. The choice of these particular ions was dictated by the immediate application of the reported sensors to measure the temperature and pH in biological tissues and cells. These ions are the most abundant in biological buffers. It should be noted that the applicability of sensors to other environments has to be tested for each specific environment. Because the number of cases in which the sensors described can be used is practically unlimited, the study of spectral stability in other buffers is beyond the scope of this work. Furthermore, if this problem appears, it could potentially be addressed by additional nonporous silica coating.

## 5. Conclusions

In the present work, we describe a dual acidity (pH) and temperature sensor based on ultrabright fluorescent nanoparticles. Simultaneous, multiparametric and nonlinear pH and temperature sensing were successfully demonstrated using FITC (a pH-sensitive dye), rhodamine B (a temperature-sensitive dye) and Nile blue (a reference dye) physically mixed in mesoporous silica particle.

To measure both pH and temperature simultaneously, using two ratios of fluorescent intensities measured at four wavelengths was suggested. A regression analysis was used to find regions of four wavelengths that are (almost) linear with pH and temperature. This allowed us to find the wavelengths to optimize the signal-to-noise ratio and reduce cross-correlation between acidity and temperature dependencies, which in turn decreased the error of the measurements when using the sensors. The regression analysis and algorithm described may further be used in multiparametric systems or sensors in which the parameters are cross-correlated and there is a need to reduce the cross-correlation. The analysis of the discrepancy between the equations and experimentally measured pH and temperature showed that the method suggested can be used to measure pH and temperature with an accuracy of greater than 98%. The repeatability of measuring pH and temperature was demonstrated by multiple cycling of temperature and acidity. Finally, the stability of the sensor in the presence of different ions typical for biological media was demonstrated. Due to a high brightness, sensing with ultrabright nanoparticles could be done at a low dosage and low intensity of the excitation light. When applied to biomedical detection, this will decrease not only the chemotoxicity but also the phototoxicity.

## Figures and Tables

**Table 1:** Fluorescence properties of ultrabright pH and temperature-sensing nanoparticles. Relative fluorescence brightness of ultrabright mesoporous silica nanoparticles in both MESF units (relative to one free dye molecule) and M^−1^ cm^−1^, and the number of dye molecules per particle. Note that all the calculations were performed using the number weighted average particle size of 51 nm.

Contributing Dye	Excitation	Emission Range	Absolute Brightness (M^−1^ cm^−1^)	Relative Brightness (MESF Units)	Number of Dye Molecules Per Particle	Volume Per One Dye Molecule (nm^3^)
FITC	490 nm	500–700 nm	(10 ± 1) × 10^6^	150 ± 10	178 ± 12	390
Rb	550 nm	560–700 nm	(12 ± 1) × 10^6^	387 ± 30	385 ± 30	180
Nb	630 nm	640–700 nm	(40 ± 3) × 10^6^	1965 ± 135	2475 ± 170	28

**Table 2:** Fitted coefficients used in Equations (4) and (5) when using the optimal wavelengths of 525, 537, 581 and 611 nm.

Coefficients for Equation (4)	Value	Coefficients for Equation (5)	**Value**
At	51,470	ApH	2498
Bt	−166,817	BpH	−8089
Ct	180,264	CpH	8747
Dt	−91.5	DpH	1.04
Et	−64,712	EpH	−3156

## Supplementary Materials

The following are available online at https://www.mdpi.com/article/10.3390/nano11061524/s1, Figure S1: Repeatability of measurements at (a and b) constant pH and different temperature and at (c and d) constant temperature and different pH. Figure S2: (a) PCC of pH when PCC of pH is greater than 0.7 while PCC of temperature is less than 0.5, PCC of temperature when PCC of temperature is greater than 0.95 while PCC of temperature is less than 0.5 and (b) SNR of pH when PCC of pH is greater than 0.7 while PCC of temperature is less than 0.5, (c) PCC and (d) SNR of temperature when PCC of temperature is greater than 0.95 while PCC of pH is less than 0.5. Figure S3: Temperature and pH response of nanosensors towards metal ions (1 mM) K^+^, Ca^2+^, Mg^2+^ and Na^+^. Table S1: Ratios of fluorescence intensity at wavelengths of 525 and 537 nm (RpH) and 581 and 611 nm (RT) for measuring pH and temperature. Error represents the standard deviation of four measurements. Table S2: Temperature and standard deviation calculated using Equation (4) (considering pH fixed). Both temperature and pH in the table headings are measured using standard tools (a thermocouple and pH meter). Table S3: pH and standard deviation calculated using Equation (5) (considering temperature fixed). Table S4: FRET efficiency and distance between different dye molecules. Both temperature and pH in the table headings are measured using standard tools (a thermocouple and pH meter). Table S5: Repeatability of the sensor ratios.
