# Supplementary material for: Ultrabright Fluorescent Silica Nanoparticles for Dual pH and Temperature Measurements
Source: Nanomaterials (Basel). 2021 Jun 9;11(6):1524. doi: 10.3390/nano11061524 (PMC8228773; doi:10.3390/nano11061524)
Supplement: Supplementary file 1 [file nanomaterials-11-01524-s001.zip › nanomaterials-1243339-supplementary.pdf]

nanomaterials

Supplementary Information

Ultrabright Fluorescent Silica Nanoparticles for Dual pH and Temperature Measurements

Saquib Ahmed M. A. Peerzade [1], Nadezhda Makarova [2] and Igor Sokolov [1,2,3,*]

[1] Department of Biomedical Engineering, Tufts University, Medford, MA 02155, USA; Saquib.Peerzade@tufts.edu
[2] Department of Mechanical Engineering, Tufts University, Medford, MA 02155, USA; Nadezda.Makarova@tufts.edu
[3] Department of Physics, Tufts University, Medford, MA 02155, USA
* Correspondence: igor.sokolov@tufts.edu; Tel.: +1 (617)-627-2548

1. Calculation of the Pearson Correlation Coefficient

The Pearson correlation coefficient is calculated separately for pH and temperature dependencies. For example, this coefficient calculated for the dependence of pH, $\mathbf{r^2_{pH}}$ where, R is the ratio of fluorescence intensities at two wavelengths, is given by:

$$r^2_{pH} = \frac{S^2_{pH,R}}{S_{pH}S_R}, \tag{S1}$$

where, S_{pH} and S_R are standard deviations of pH and R respectively, and $S_{pH,R}$ is the covariance. The standard deviation of pH is given by,

$$S_{pH} = \sum_{pH_1}^{pH_n} \frac{(pH - pH_{mean})^2}{n_{pH}} \tag{S2}$$

where, $pH_{1\ to\ n}$ are the pH values used in the measurements (6.8, 6.3, 5.8, 5.3, 4.8 and 4.3), pH_{mean} is the mean of all the pH values and n_{pH} are the total number of the pH measurements.

The standard deviation of *R* is given by,

$$S_R = \sum_{pH_1}^{pH_n} \frac{\left(\sum_{T_1}^{T_n}\left(\frac{R}{n_T}\right) - \overline{R}\right)^2}{n_{pH}} \tag{S3}$$

where, $T_{1\ to\ n}$ are the temperature values 25, 30, 35, 40 and 45 ˚C respectively used in the calculation, n_T are the total number of the temperature measurements and $\mathbf{\overline{R}}$ is the average ratio across all pH (and temperatures) given by,

$$\overline{R} = \frac{1}{n_{pH}} \sum_{pH_1}^{pH_n} \sum_{T_1}^{T_n} \left(\frac{R}{n_T}\right) \tag{S4}$$

The covariance of pH and R is given by,

$$S_{pH,R} = \sum_{pH_1}^{pH_n} \left(\frac{(pH - pH_{mean}) \left(\sum_{T_1}^{T_n} \left(\frac{R}{n_T} \right) - \bar{R} \right)}{n_{pH}} \right) \tag{S5}$$

Similarly, the Person coefficient for the temperature function T(R) can be calculated. The standard deviation of temperature (S_T) is given by,

$$S_T = \sum_{T_1}^{T_n} \frac{(T - T_{mean})^2}{n_T} \tag{S6}$$

where, T_{mean} is the mean of all the temperature values.

The standard deviation of R is given by,

$$S_R = \sum_{T_1}^{T_n} \left(\frac{\left(\sum_{pH_1}^{pH_n} \left(\frac{R}{n_{pH}} \right) - \bar{R} \right)^2}{n_T} \right) \tag{S7}$$

where, $\bar{\mathbf{R}}$ is the average ratio across all temperatures (and pH) given by,

$$\bar{R} = \frac{1}{n_T} \sum_{T_1}^{T_n} \sum_{pH_1}^{pH_n} \left(\frac{R}{n_{pH}} \right) \tag{S8}$$

The covariance of T and R is given by,

$$S_{T,R} = \sum_{T_1}^{T_n} \left(\frac{(T - T_{mean}) \left(\sum_{pH_1}^{pH_n} \left(\frac{R}{n_{pH}} \right) - \bar{R} \right)}{n_T} \right) \tag{S9}$$

2. Variance Formulae

$$S(t) = \left| \frac{\left(\left(\frac{I_1}{I_2}\right)_{T1} - \left(\frac{I_1}{I_2}\right)_{T2} \right)}{\left(\frac{\left(\frac{I_1}{I_2}\right)_{T1} + \left(\frac{I_1}{I_2}\right)_{T2}}{2} \right)} \right| = \left| \frac{(A-B)}{\left(\frac{A+B}{2}\right)} \right| = \left| \frac{(1-B/A)}{\left(\frac{1+B/A}{2}\right)} \right| = \left| 2 * \frac{\left(1-\frac{B}{A}\right)}{\left(1+\frac{B}{A}\right)} \right| = \left| \frac{2*t}{(2-t)} \right| = \left| \frac{2}{(2/t-1)} \right|, \tag{S10}$$

$$t(A, B) = 1 - B/A$$

$$\boldsymbol{\delta t(A, B) = \sqrt{(\partial_A t)^2 (\delta A)^2 + (\partial_B t)^2 (\delta B)^2}}$$

$$\boldsymbol{= \sqrt{(\partial_A (1 - B/A))^2 (\delta A)^2 + (\partial_B (1 - B/A))^2 (\delta B)^2}}$$

$$\boldsymbol{\delta t = \sqrt{\frac{B^2 (\delta A)^2 + A^2 (\delta B)^2}{A^4}}}$$

$$\boldsymbol{\delta S(t)} = \sqrt{(\partial_t S)^2(\delta t)^2} = \delta t(A,B) = \sqrt{\left(\partial_t \frac{2}{\left(\frac{2}{t}-1\right)}\right)^2 (\delta t)^2} = 4\sqrt{\frac{(\delta t)^2}{(t-2)^4}}$$

$$\boldsymbol{\delta S(A,B)} = 4\sqrt{\frac{\left(\sqrt{\frac{B^2(\delta A)^2 + A^2(\delta B)^2}{A^4}}\right)^2}{((1-B/A)-2)^4}} = 4\sqrt{\frac{B^2(\delta A)^2 + A^2(\delta B)^2}{(A+B)^4}}$$

3. Calculating Error in Temperature and pH Measurements

Using the error propagation formula, the error in temperature T ($\mathbf{R_T}$, $\mathbf{R_{pH}}$) is given by,

$$\Delta T(R_T, R_{pH}) = \sqrt{\sigma R_{pH}{}^2 * \left(\frac{\partial T(R_T, R_{pH})}{\partial R_{pH}}\right)^2 + \sigma R_T{}^2 * \left(\frac{\partial T(R_T, R_{pH})}{\partial R_T}\right)^2}, \quad \text{(S11)}$$

$$\Delta T(R_T, R_{pH}) = \sqrt{\sigma R_{pH}{}^2 \left(3AtR_{pH}{}^2 + 2BtR_{pH} + Ct + DtR_T\right)^2 + Dt^2 R_{pH}{}^2 \sigma R_T{}^2}, \quad \text{(S12)}$$

where, $\boldsymbol{\sigma R_T}$ and $\boldsymbol{\sigma RpH}$ are the standard deviations of 4 measurements of ratios $\mathbf{R_T}$ and **RpH** respectively. Similarly, the error in pH (pH ($\mathbf{R_T}$, $\mathbf{R_{pH}}$)) is given by,

$$\Delta pH(R_T, R_{pH}) = \sqrt{\sigma R_{pH}{}^2 * \left(\frac{\partial pH(R_T, R_{pH})}{\partial R_{pH}}\right)^2 + \sigma RT^2 * \left(\frac{\partial pH(R_T, R_{pH})}{\partial R_T}\right)^2}, \quad \text{(S13)}$$

$$\Delta pH(R_T, R_{pH}) = \sqrt{\sigma R_{pH}{}^2 \left(3ApHR_{pH}{}^2 + 2BpHR_{pH} + CpH + DpHR_T\right)^2 + DpH^2 R_{pH}{}^2 \sigma R_T{}^2}. \quad \text{(S14)}$$

Table S1. Ratios of fluorescence intensity at wavelengths of 525 and 537 nm (R_{pH}) and 581 and 611 nm (R_T) for measuring pH and temperature. Error represents the standard deviation of 4 measurements.

Known T (°C)	Known pH	R_{pH} = I(525)/I(537)	R_T = I(581)/I(611)
24.17	6.8	1.133 ± 0.001	2.138 ± 0.003
24.17	6.3	1.126 ± 0.003	2.109 ± 0.006
24.17	5.8	1.113 ± 0.001	2.096 ± 0.004
24.17	5.3	1.092 ± 0.004	2.111 ± 0.006
24.17	4.8	1.063 ± 0.005	2.147 ± 0.022
24.17	4.3	1.043 ± 0.007	2.168 ± 0.004
29.63	6.8	1.138 ± 0.002	2.091 ± 0.006
29.63	6.3	1.132 ± 0.002	2.067 ± 0.002
29.63	5.8	1.116 ± 0.001	2.044 ± 0.010
29.63	5.3	1.091 ± 0.002	2.080 ± 0.032
29.63	4.8	1.066 ± 0.002	2.098 ± 0.007
29.63	4.3	1.041 ± 0.003	2.103 ± 0.003
34.74	6.8	1.139 ± 0.002	2.054 ± 0.006
34.74	6.3	1.132 ± 0.002	2.029 ± 0.013
34.74	5.8	1.117 ± 0.001	1.995 ± 0.010
34.74	5.3	1.093 ± 0.004	2.012 ± 0.006
34.74	4.8	1.064 ± 0.004	2.039 ± 0.011
34.74	4.3	1.041 ± 0.006	2.041 ± 0.007
39.77	6.8	1.143 ± 0.002	2.015 ± 0.012

39.77	6.3	1.138 ± 0.003	1.993 ± 0.007
39.77	5.8	1.122 ± 0.002	1.955 ± 0.006
39.77	5.3	1.093 ± 0.001	1.962 ± 0.009
39.77	4.8	1.066 ± 0.002	1.986 ± 0.014
39.77	4.3	1.040 ± 0.002	1.991 ± 0.017
44.80	6.8	1.144 ± 0.002	1.976 ± 0.008
44.80	6.3	1.139 ± 0.001	1.952 ± 0.004
44.80	5.8	1.123 ± 0.001	1.916 ± 0.004
44.80	5.3	1.092 ± 0.003	1.920 ± 0.017
44.80	4.8	1.065 ± 0.001	1.936 ± 0.009
44.80	4.3	1.043 ± 0.008	1.934 ± 0.015

Table S2. Temperature and standard deviation calculated using Equation (4) (considering pH fixed). Both temperature and pH in the table headings are measured using standard tools (a thermocouple and pH meter).

pH / T (°C)	6.8	6.3	5.8	5.3	4.8	4.3
**24.17**	23.2 ± 0.2	24.6 ± 0.7	24.7 ± 0.5	24.7 ± 0.8	25.1 ± 1.9	23.7 ± 0.6
**24.17**	29.4 ± 0.7	30.1 ± 0.7	30.2 ± 1.2	29.2 ± 1.8	29.6 ± 0.8	29.8 ± 0.5
**24.17**	34.1 ± 0.8	34.3 ± 1.8	35.2 ± 1.1	34.5 ± 0.3	35.5 ± 1.0	35.6 ± 1.1
**24.17**	39.5 ± 2.0	39.8 ± 1.5	39.7 ± 0.7	39.5 ± 1.1	40.6 ± 1.2	40.5 ± 1.9
**24.17**	44.2 ± 1.0	44.3 ± 0.5	44.0 ± 0.5	43.8 ± 1.8	45.5 ± 0.8	45.9 ± 1.4

Table S3. pH and standard deviation calculated using Equation (5) (considering temperature fixed). Both temperature and pH in the table headings are measured using standard tools (a thermocouple and pH meter).

pH / T (°C)	6.8	6.3	5.8	5.3	4.8	4.3
**24.17**	6.51 ± 0.04	6.21 ± 0.07	5.81 ± 0.04	5.37 ± 0.07	4.90 ± 0.08	4.46 ± 0.22
**29.63**	6.64 ± 0.07	6.37 ± 0.05	5.84 ± 0.04	5.32 ± 0.04	4.90 ± 0.03	4.33 ± 0.11
**34.74**	6.67 ± 0.10	6.35 ± 0.07	5.82 ± 0.02	5.28 ± 0.06	4.80 ± 0.06	4.26 ± 0.13
**39.77**	6.77 ± 0.08	6.54 ± 0.11	5.90 ± 0.04	5.23 ± 0.01	4.77 ± 0.05	4.19 ± 0.07
**44.8**	6.80 ± 0.10	6.52 ± 0.07	5.90 ± 0.02	5.15 ± 0.10	4.70 ± 0.04	4.19 ± 0.19

4. Calculating the fluorescence brightness of the nanosensor

The brightness of particles relative to the dye (MESF units) as shown in Table 1 is given by,

$$\text{Relative Brightness} = \frac{\mathrm{IF}_{\text{particles}} / \text{Number of particles}}{\mathrm{IF}_{\text{Dye}} / \text{Number of dye molecules}} \quad \text{(S15)}$$

The QY of the particles is given by,

$$\Phi_{\text{particles}} = \Phi_{\text{Dye}} \left(\frac{\frac{\mathrm{IF}_{\text{Particles}}}{\mathrm{A}_{\text{Dye extracted from particles}}}}{\frac{\mathrm{IF}_{\text{Dye}}}{\mathrm{A}_{\text{Dye}}}} \right) \left(\frac{\eta_{\text{Particles}}{}^{2}}{\eta_{\text{Dye}}{}^{2}} \right) \quad \text{(S16)}$$

where, $\Phi_{\text{particles}}$ is the quantum yield of the particles, Φ_{Dye} is the QY of dye, $\eta_{\text{particles}}$ and η_{Dye} are the refractive index of solvent (water) into which the particles and dye were added respectively.

QY of particles can also be calculated using the equation,

$$\Phi_{particles} = \Phi_{Dye}\left(\frac{\text{Relative brightness in MESF units}}{\text{Number of dye molecules per particle}}\right) \quad (S17)$$

It should be noted that there is another well-known method of calculation of the fluorescent brightness. The brightness of the particles can be calculated as follows [1].

$$B = \varepsilon_{particles}(\lambda) \times \phi_{particles}, \quad (S18)$$

Table S4. FRET efficiency and distance between different dye molecules.

Dye molecules	Number of dye molecules per particle	Distance between dye molecules	R_0	FRET Efficiency
FITC & RB	563 ± 30	5.3 ± 0.1	5.5[2]	0.56 ± 0.02
RB & NB	2860 ± 170	3.08 ± 0.06	5.4[3]	0.97 ± 0.004

5. Repeatability of pH and Temperature Measurements

Repeatability of the described sensors is shown in Figure S1. Fluorescent spectra from nanosensors were measured at pH 7 for temperature 35 and 50 °C and similarly, at pH 4 for temperatures 35 and 50 °C for 4 cycles of temperature. Table below shows the ratios at different pH and temperature. The error is defined as the standard deviation across fours measurement cycles shown in Figure S1. One can see that the error to signal ratio is less than 1%.

Table S5. Repeatability of the sensor ratios.

T (°C)	pH	I(525nm)/I(537nm)	I(581nm)/I(611nm)
35	7	1.172 ± 0.002	2.323 ± 0.016
35	4	1.052 ± 0.004	2.159 ± 0.018
50	7	1.181 ± 0.001	2.174 ± 0.007
50	4	1.050 ± 0.006	1.969 ± 0.019

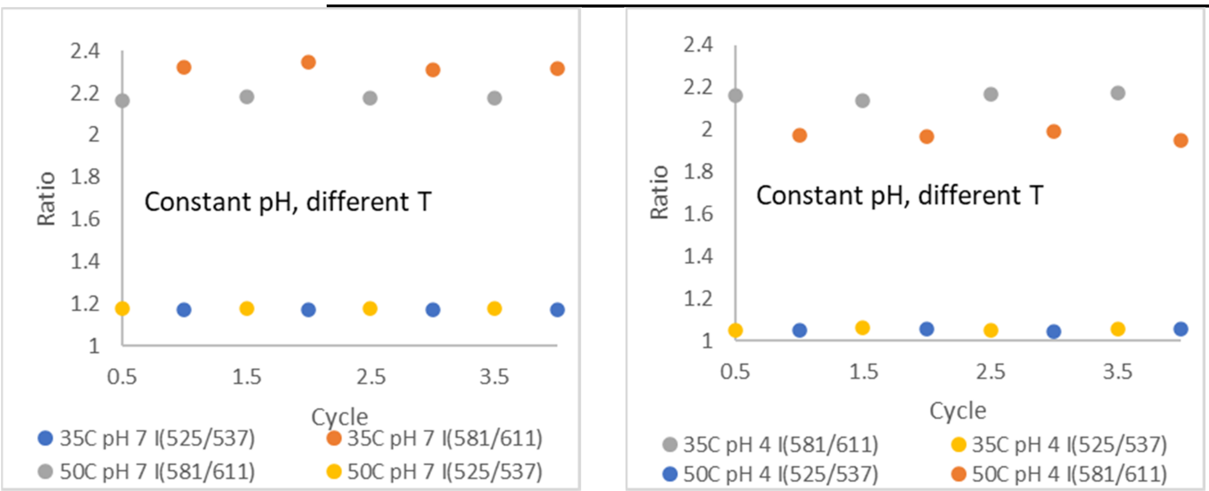

(**a**) (**b**)

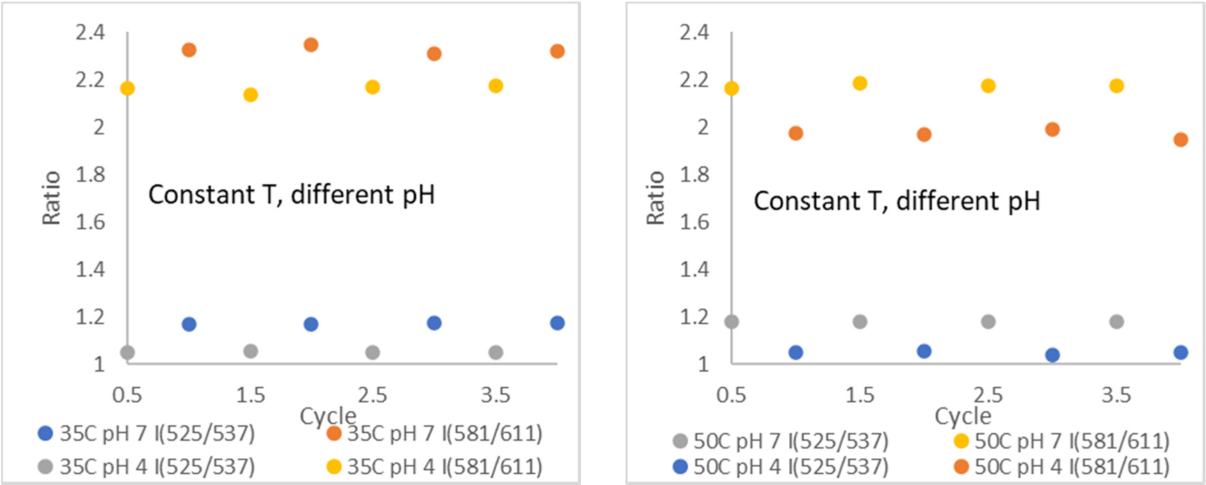

(**c**) (**d**)

Figure S1. Repeatability of measurements at **(a,b)** constant pH and different temperature and at **(c,d)** constant temperature and different pH.

6. Finding the Optimal Values of Wavelengths to Measure Temperature and Acidity Simultaneously

Figure S2 shows the PCC for pH (Figure S2a) and SNR (Figure S2c) for pH for all the wavelengths from 500–650 nm for PCC of pH greater than 0.7 and PCC of temperature less than 0.5. Also, the PCC for temperature (Figure S2b) and SNR (Figure S2d) for pH for all the wavelengths from 500–650 nm for PCC of temperature greater than 0.95 and PCC of temperature less than 0.5. Since for the wavelength range 500-550nm the SNR for pH is high when PCC for pH is greater than 0.7 and PCC for temperature is less than 0.5, PCC and SNR for pH in this wavelength range is shown in Figure. 6c and e respectively. And since for the wavelength range 560–650 nm the SNR for temperature is high when PCC for temperature is greater than 0.95 and PCC for temperature is less than 0.5, PCC and SNR for temperature in this wavelength range is shown in Figure 6d,f respectively.

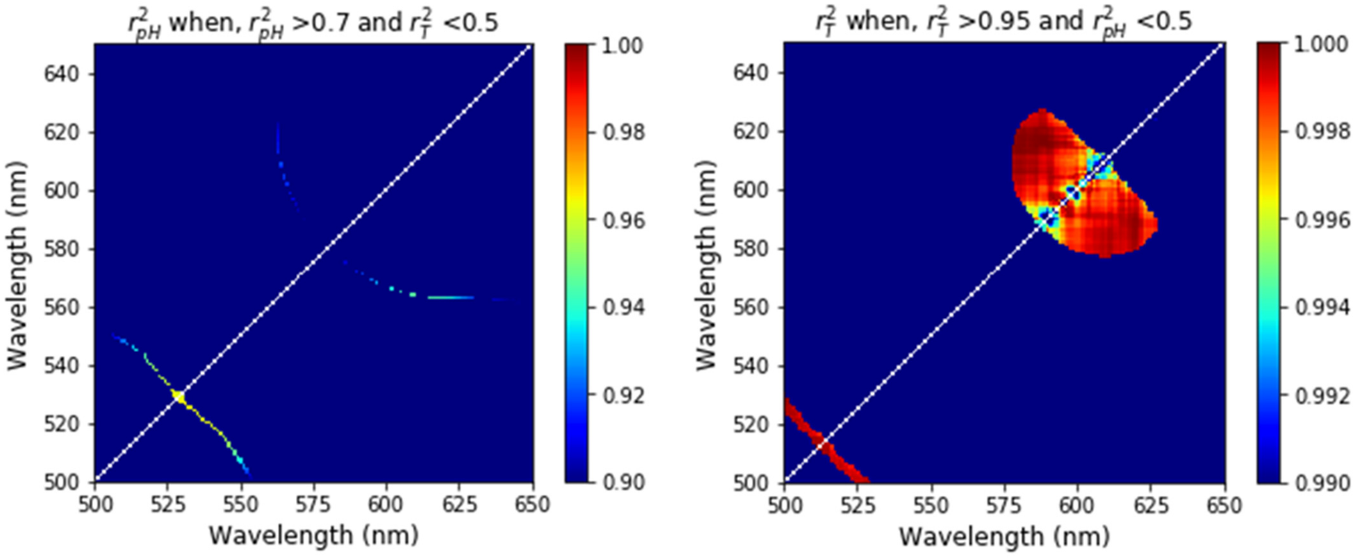

(**a**) (**b**)

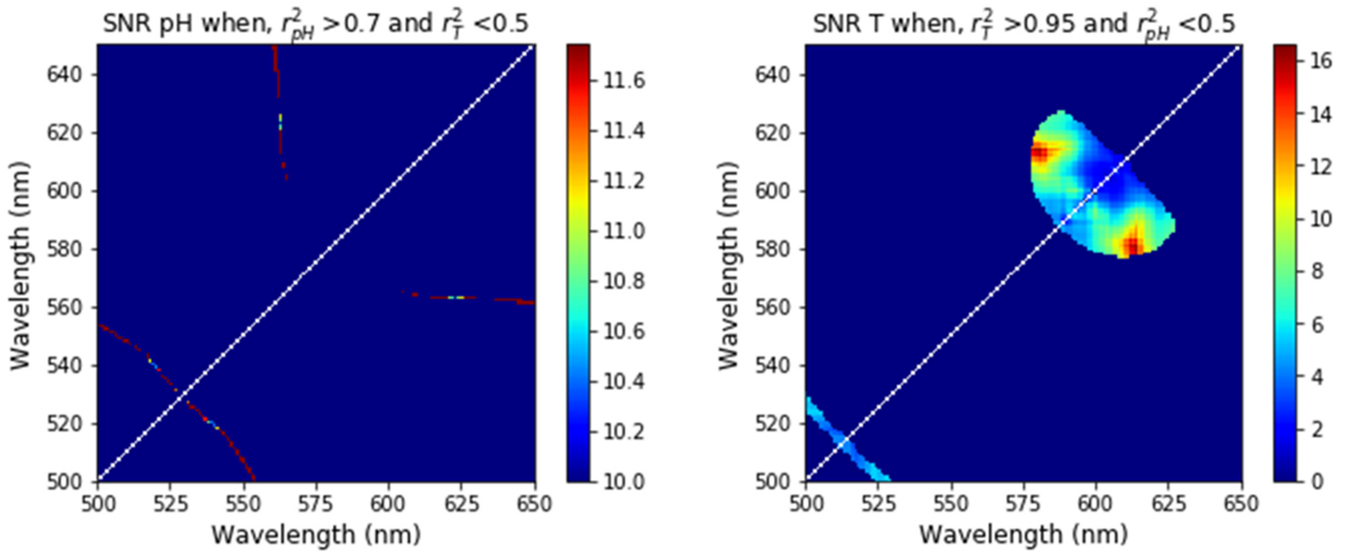

(**c**) (**d**)

Figure S2. **(a)** PCC of pH when PCC of pH is greater than 0.7 while PCC of temperature is less than 0.5, PCC of temperature when PCC of temperature is greater than 0.95 while PCC of temperature is less than 0.5 and **(b)** SNR of pH when PCC of pH is greater than 0.7 while PCC of temperature is less than 0.5, **(c)** PCC and **(d)** SNR of temperature when PCC of temperature is greater than 0.95 while PCC of pH is less than 0.5.

7. Stability in Presence of Ions

Figure S3 shows differently the data presented in the main text in Figure. 3. The current presentation might be more convenient for some readers.

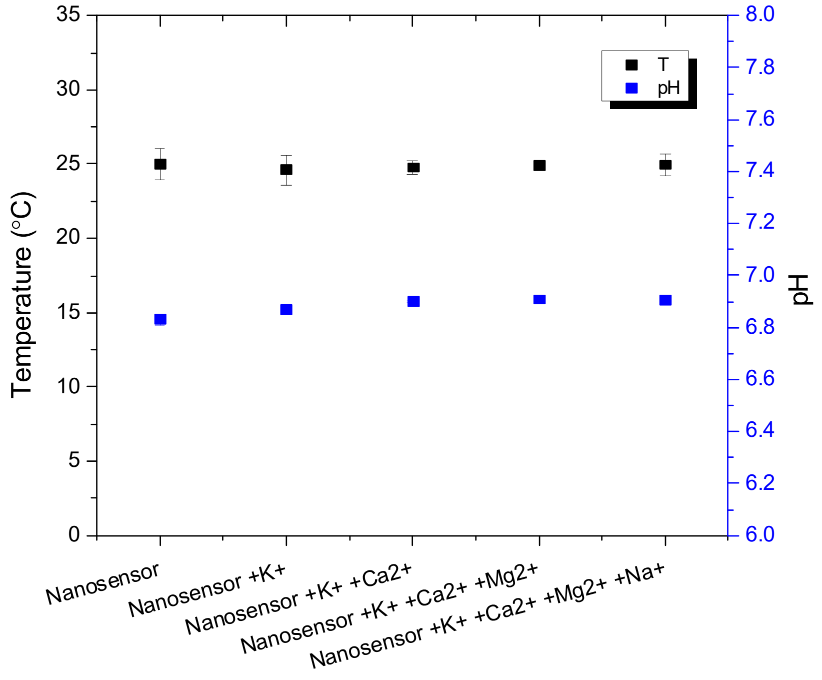

^

Figure S3. Temperature and pH response of nanosensors towards metal ions (1mM) K^+, Ca^{2+}, Mg^{2+} and Na^+.

References

1. Lim, S.J.; Zahid, M.U.; Le, P.; Ma, L.; Entenberg, D.; Harney, A.S.; Condeelis, J.; Smith, A.M. Brightness-equalized quantum dots. *Nat. Commun.* **2015**, *6*, 8210.
2. Chigaev, A.; Smagley, Y.; Haynes, M.K.; Ursu, O.; Bologa, C.G.; Halip, L.; Oprea, T.; Waller, A.; Carter, M.B.; Zhang, Y. FRET detection of lymphocyte function–associated antigen-1 conformational extension. *Mol. Biol. Cell* **2015**, *26*, 43–54.
3. Lu, P.; Yu, Z.; Alfano, R.; Gersten, J. Picosecond studies of energy transfer of donor and acceptor dye molecules in solution. *Phys. Rev. A* **1982**, *26*, 3610.
